# Participants' Experiences of Being Treated for Peri‐Implant Mucositis—A Qualitative Interview Study

**DOI:** 10.1111/idh.12847

**Published:** 2024-10-14

**Authors:** Viveca Wallin Bengtsson, Kirsti Skovdahl, Pia Andersson

**Affiliations:** ^1^ Department of Oral Health, Faculty of Health Sciences Kristianstad University Kristianstad Sweden; ^2^ Faculty of Health, Welfare and Organization Østfold University College Halden Norway

**Keywords:** Er:YAG laser, participant experiences, peri‐implant mucositis, person centredness, qualitative interview, ultrasonic scaler

## Abstract

**Objective:**

To describe participants' experiences of being treated for peri‐implant mucositis.

**Methods:**

A qualitative study with nine individual, semistructured interviews was performed. The interview guide was based on a focus group interview. The participants had recently been treated for peri‐implant mucositis on one dental implant in a randomised controlled trial (RCT). The treatment included information, oral hygiene instructions, nonsurgical treatment with Er:YAG laser or ultrasonic scaler and professional cleaning in several sessions over 6 months. The interviews performed were analysed using qualitative manifest and latent content analysis.

**Results:**

The manifest results showed that learning how to brush the teeth, and repeated feedback, was appreciated, and increased the motivation to improve oral hygiene habits. Most participants experienced no discomfort from the treatment. The participants had an understanding that a longer treatment time was required to ensure the quality of the treatment. Respect and attention were important elements of the personal treatment. The latent results suggest that treatment with a laser or an ultrasonic scaler was not perceived as the most important part of the treatment. Participants felt that receiving information about the treatment process was more important; moreover, a person‐centred approach gave a feeling of good and safe care.

**Conclusions:**

The present study highlights factors of importance in treatment of peri‐implant mucositis with laser and ultrasonic scaler. A person‐centred approach with respect and attention is important for a good and safe experience and may be important factors in future treatments.

## 1 Introduction

Dental implants have, in the last four decades, become a treatment option to replace missing teeth [1]. Plaque accumulation may induce an inflammation in the soft tissues surrounding the implant, causing so‐called ‘peri‐implant mucositis’ [2, 3, 4]. Prevalence of peri‐implant mucositis has been reported in 80% of patients, and on an implant level in 50% [5]. If not treated, peri‐implant mucositis may progress to peri‐implantitis, with subsequent bone loss and failure of the dental implant, and with negative consequences for the individual affected [6, 7].

When effectively treated, peri‐implant mucositis is reversible [2, 8]. The objective of peri‐implant mucositis treatment is primarily to alter the microbiota so that it will become compatible with the host to allow healing and a clinically healthy situation. Nonsurgical treatment is critical and is effective if the patient maintains a satisfactory level of oral hygiene [7, 9]. Enough time to ensure that oral hygiene instructions are properly delivered, and that the patient understands the importance of effective plaque control, is a prerequisite [10, 11]. Nonsurgical treatment can be performed by specially designed hand instruments or ultrasonic scaler with plastic or Teflon‐coated tips. Nonsurgical adjunctive therapies such as antibiotics, antiseptics and laser treatments have been studied [12]. Treatment with laser therapy, for example, Er:YAG laser, should be considered a minimally invasive technique and may result in less discomfort than caused by traditional treatment approaches. Furthermore, lasers may have a biostimulatory effect and are reported to result in better wound healing, compared with traditional approaches, as well as in periodontal tissue regeneration [13].

In patients undergoing peri‐implant maintenance care, 70.4% agreed or strongly agreed in a questionnaire that implants are a treatment providing a solution for the rest of life. Most patients receiving implants had no knowledge about peri‐implant pathology [14].

Patient‐reported outcome measures (PROMs) have infrequently been described in studies on prevention and management of peri‐implant mucositis [15]. In a qualitative interview study, it was shown that patients initially had very high expectations of dental implant therapy, hoping it might permanently solve their dental problems [16].

Only a few qualitative studies exist that are based on interviews exploring patients' experiences of undergoing treatment for peri‐implantitis [16, 17]. As far as we know, no study has thus far investigated patients' experience of being treated for peri‐implant mucositis. Therefore, the aim of this study was to describe the participants' experiences of being treated for peri‐implant mucositis.

## 2 Materials and Methods

A qualitative, explorative study was conducted to gain a deeper understanding of the participants' experiences and needs related to being treated for peri‐implant mucositis. The Standards for Reporting Qualitative Research (SRQR) checklist and the Consolidated Criteria for Reporting Qualitative Research (COREQ) checklist were followed (Data S1 and S2).

### 2.1 Recruitment of the Participants

Nine participants were recruited from a clinical study in which patients were referred to a Specialty Clinic of Periodontics in Sweden for inclusion in a randomised controlled trial (RCT) treating peri‐implant mucositis on one dental implant (in manuscript). In the RCT, the dental implants underwent treatment with either an erbium–yttrium aluminium garnet (Er:YAG) laser or an ultrasonic scaler by a periodontist. The treatment was limited to four sessions in 6 months. In both groups, oral hygiene instructions were included and reinforced at each session by both the periodontist and a dental hygienist. The treatment for peri‐implant mucositis consisted of information about disease origin, pathogenesis and therapy, oral hygiene instructions, treatment with laser or ultrasonic scaling and professional cleaning, and was carried out in the same way with each participant.

All patients in this study are referred to as ‘participants’. To ensure heterogeneity and diversity, the participants were strategically selected by the first author (V.W.B.) for the interviews, with consideration to age, gender and good oral communication skills, after being treated for peri‐implant mucositis. First an initial focus group interview was performed to develop an interview guide, in which five out of five invited participants (two women and three men) were contacted by phone and agreed to participate. Thereafter 10 more participants were contacted by phone and informed about the aim of an individual interview and invited to participate. One out of 10 individuals declined because of time pressure. The study sample consisted of nine participants (four women and five men), mean age 68.2 years, range 51–76 years (Table 1).

**TABLE 1:** Characteristics of the study participants.

Age (years)	Gender, F/M	Treatment, Er:YAG/Ultrasonic scaler	ID number in interview
65	F	Ultrasonic scaler	1
76	M	Ultrasonic scaler	2
75	M	Er:YAG	3
75	M	Er:YAG	4
69	F	Er:YAG	5
68	F	Er:YAG	6
71	F	Er:YAG	7
64	M	Ultrasonic scaler	8
51	M	Er:YAG	9

*Note:* Er:YAG is erbium–yttrium aluminium garnet (laser) with tip PS400TS. Ultrasonic scaler with tip PI Instrument Nyon, EMS, Switzerland.

Abbreviations: F = female; M = male.

### 2.2 Procedure

#### 2.2.1 Focus Group Interview

Initially, five of the study participants were invited to attend a focus group interview 0.5–5.0 months (mean 3.5) after finalising their treatment in the RCT. Based on their experiences of participating in the RCT study, the informants were asked to give input on questions that they felt were important to raise in an interview guide which should be used in the individual interviews. Information gained from the focus group interview was not a part in the analysis and result but was only used for the design of the interview guide.

The first author (V.W.B.) participated in the dialogue, while the second author (K.S.) observed, took field notes and asked additional questions if needed. The participants were encouraged to actively and as freely as possible discuss different experiences and views of being treated for peri‐implant mucositis. Through the use of focus group methodology and listening to the participants' opinions, new insights were gained [18]. The interviews were tape‐recorded and transcribed verbatim. Thereafter an interview guide was developed based on the participants' inputs and in line with the aim of the study. The focus group interview lasted 65 min and was conducted in September 2022 in a group room in a neutral place outside the dental clinic. As a member check the suggested interview guide was read by one of the participants in the focus group and amended to ensure that the questions were understandable.

#### 2.2.2 Individual Interviews

Individual semistructured interviews were conducted 0.5–6.0 months (mean 2.8) after finalising the treatments, in the same place as the focus group by the first author (V.W.B.) and guided by the interview guide. The individual interviews were performed 1.0–2.5 months (mean 2.1) after the focus group interview. The interview guide was pilot tested with two of the participants. These interviews were of high quality and have, therefore, been included in the data material.

The interview guide covered different themes related to the participants' experience of the treatment of peri‐implant mucositis (Table 2). The interview started with the question: ‘You have been treated for inflammation around a dental implant—how have you experienced the treatment?’ Based on the answers, follow‐up and probing questions were asked to cover the themes and to get as much information as possible in relation to the aim of the study. Throughout the data collection process, data saturation was discussed within the research team. Data saturation was considered to have been reached after nine interviews as no new information was added in the last two interviews. The interviews were conducted from September to October 2022 and lasted between 19 and 45 min, with a mean of 28 min. All interviews were tape‐recorded with permission from the participants.

**TABLE 2:** Interview guide with seven themes.

Themes	Description
1	Experiences of the oral hygiene instructions
2	Experiences of the personal treatment
3	Experiences of how disease information has affected knowledge
4	Experiences of the time required for the treatment
5	Experiences of impact on everyday life
6	Experiences of impact on oral appearance
7	Experiences of impact on quality of life

### 2.3 Analysis

The interviews were transcribed verbatim and analysed using qualitative manifest and latent content analysis as described by Graneheim and Lundman [19]. All authors collaborated in the analysis of the interviews. Two of the authors (K.S. and P.A.) had experience in conducting qualitative interview studies.

The entire text was read carefully several times to get an overall overview of the material. In the next step, the text content was divided into meaning units relevant to the aim of the study. Later these meaning units were condensed and coded. The codes were compared to identify similarities and differences, and grouped into subcategories and, finally, categories. The categories and subcategories were refined, discussed and negotiated within the research team. Subcategories with a similar meaning were consolidated into a category. Three categories describing experiences and perspectives of being treated for peri‐implant mucositis were developed in the manifest phase. In the latent step, the text was reflected on and the question ‘What are these interviews about?’ was asked. An overall theme was constructed that covered the interpretation of the underlying meaning of the data [19].

### 2.4 Ethical Considerations

The National Ethics Review Board in Sweden approved a supplementary protocol for the interview study (2022‐03487‐02_A). The World Medical Association Declaration of Helsinki was followed in the conduct of the study. The participants were given oral information about the interview and had the opportunity to ask questions. They were informed of the right to withdraw from the study without needing to give a reason. For confidentiality all personal information was removed and the participants were given a code number. The code list and all data including the tape‐recorded files were saved according to recommendations from the university.

## 3 Results

In the following text, the findings will be presented category by category and illustrated with interview quotations. The main theme ‘Comprehensive care as an important factor for a good and safe treatment experience’, emerged in the latent phase of the analysis. This theme is built on the three categories ‘Experience of having learnt something new’, ‘Sensory and emotional experience of the treatment process’ and ‘Experience of quality and safe care in the treatment’ (Table 3). The categories emerged in the manifest phase of the analysis.

**TABLE 3:** Main theme, categories, subcategories and examples of codes from the content analysis of the participants' experience of being treated for peri‐implant mucositis.

Main theme	Categories	Subcategories	Codes
Comprehensive care as an important factor for a good and safe treatment experience	Experience of having learnt something new	The meaning of treatment information	A need for information
			Increased knowledge
			Received information of all treatment steps
		Changed oral hygiene habits	Feedback
			Instruction including practical training
			Oral hygiene
		Motivation for self‐care	Careless
			Cleaner after oral hygiene instruction
			Fresher in the mouth
			Keep dental status
	Sensory and emotional experience of the treatment process	Pain or discomfort of the treatment process	Discomfort with things in the mouth
			Increased impact of pain
			Neither pain nor discomfort
			Pain or discomfort after the treatment
			Pain or discomfort as a result of the treatment
		Intraoral changes of the treatment process	Better breath
			Reduced bleeding
			Paler gums
	Experience of quality and safe care in the treatment	Personal treatment	Attention
			Careful work
			Respectful
		A positive treatment result requires time	Time‐consuming
			Understanding of the time needed

### 3.1 Experience of Having Learnt Something New

This category is based on three subcategories. ‘The meaning of treatment information’, ‘Changed oral hygiene habits’ and ‘Motivation for self‐care’.

#### 3.1.1 The Meaning of Treatment Information

The participants emphasised that there is a need for information about what will happen during the treatment sessions. They felt that information in advance was important as this enabled them to know what to expect and it made it easier to relax, and thus reduced the treatment discomfort. According to the participants, this was especially important when having dental fear because they experienced that the fear was reduced by the treatment information. It was also expressed that the mouth is personal and information concerning treatment to the mouth is very important.

I want to know why I am to open my mouth as I have a personal relationship with my mouth. (#5)

Information about all steps in what was going to happen during the treatment was described as important. The participants considered the information easy to understand. They said the information was carefully given in advance already at the first telephone contact and then repeated at every appointment.

The participants described how they had gained knowledge about bacteria and the aetiology of inflammation. Knowledge about implants and that they can be troublesome was new information to them.

I've gained knowledge about implants. When I joined this study, I had no idea that implants can be troublesome. I thought that once an implant is inserted there will be no problems. So I was almost shocked when I learnt that implants can cause problems. (#1)

#### 3.1.2 Changed Oral Hygiene Habits

The participants perceived that brushing the teeth in a technically more efficient way with an electric toothbrush and interdental brushes made the mouth cleaner. They described how they had developed new habits, spent more time and focused on oral hygiene. Knowing how to take care of the teeth in a better way seemed to benefit the participants the most. Knowledge about important brushing areas such as between the gums and the teeth and about hard‐to‐reach areas such as the approximal areas was new to the participants. One participant had even never been taught the technique for using interdental brushes before.

I learned a better way to brush my teeth and how to use these interdental brushes. It's a while since I learned that about interdental brushes. In fact, never. (#8)

Some participants expressed that they really appreciated that the oral hygiene instructions were hands‐on, and said it was strange that this was not offered at the regular dental clinic.

But also, it was shown hands‐on, and we were shown when we did something wrong or something like that. This is the most efficient way I think, and this was not the case before, at the regular dental clinic. (#2)

The participants expressed that they liked to get feedback on their oral hygiene performance. It was good to first have the teeth dyed with disclosing dye to see the results, and then to be shown how to brush the teeth to get rid of the plaque. This was an easy way to see the effect of their own oral hygiene. According to some participants, it strengthened their motivation for good oral hygiene. Repetition was perceived as necessary, otherwise, the brushing tended to become perfunctory. It was also expressed that the feedback was not experienced as offensive; rather, it served as an alarm clock.

But that's what happens when you've seen that it has an effect. And the fact that you're first shown how to do it and then what effect it will have afterwards, then it's easy to take it to heart. (#2)

#### 3.1.3 Motivation for Self‐Care

Almost all participants mentioned that they felt fresher and cleaner in the mouth after the oral hygiene instructions and subsequent self‐care.

I feel it was helpful. You felt like you got a different feeling that the teeth were cleaner afterwards, when polishing; and then you want to feel that sensation with your tongue again. Yes, then you feel motivated to brush correctly. (#6)

A strong motivator for self‐care was to prevent further dental deterioration and to keep the natural teeth and the implant. In addition, professional help in oral hygiene was perceived as necessary.

It's good if you can keep all your body parts. You don't want to go toothless. (#1)

### 3.2 Sensory and Emotional Experience of the Treatment Process

This category is based on two subcategories: ‘Pain or discomfort of the treatment process’ and ‘Intraoral changes of the treatment process’.

#### 3.2.1 Pain or Discomfort of the Treatment Process

The experiences of the impact of the treatment with laser and ultrasonic scaler differed between the participants. Most participants experienced no sensation of pain or discomfort at all.

It felt very simple. It was quickly over, and it never hurt even though I'm a person with dental fear, but it was no problem at all. (#1)

A few participants experienced a sensation of discomfort during the treatment and for some the sensation lasted for several days after the treatment. An increase in the intensity of the discomfort with each treatment was also reported.

The very last treatment was more unpleasant, but not the first two treatments. The feeling was tiny though. (#6)

You could feel somebody had been there poking, hardly to the next day, then I think I forgot about it. (#3)

A sensation of discomfort at having things in the mouth such as the X‐ray sensor or the hooks was perceived by the participants.

The only thing I can come up with was when she took X‐rays of the teeth, and it was also the hooks holding up the mouth, they were not so comfortable. (#2)

#### 3.2.2 Intraoral Changes of the Treatment Process

Some participants experienced changes in the mouth after the treatment, such as no more bleeding from the gums and that the gums looked paler. It was also mentioned that the gums had moved upwards after the treatment and that less plaque was present in the photograph.

But now I haven't had it in a long time. I don't know if I ever had bleeding gums; something was [different]. Now the bleeding is almost zero. (#9)

Pale I mean the gums when you look in the mirror. But I'm not quite sure and now it looks a little bit fresher. (#9)

Someone experienced that the bad odour from the mouth that had existed before the treatment had disappeared.

Before I started this treatment it may have smelled a bit from my mouth. We have for sure noticed that it smells less after the treatment. Even my wife thinks my breath is significantly improved. (#4)

### 3.3 Experience of Quality and Safe Care in the Treatment

This category is based on two subcategories: ‘Personal treatment’ and ‘A positive treatment result requires time’.

#### 3.3.1 Personal Treatment

The personal treatment was performed with humility and some participants mentioned that they felt welcome; others, that they felt safe. The participants felt they were treated respectfully and as a person. It was expressed that they appreciated that all parts of the treatment were carefully explained. Some participants described that the intraoral treatment was personally conducted, and one participant was talking about the feeling of being on the same level as the practitioner.

The mouth is personal. It's not so easy to open your mouth for others. But this felt safe. (#5)

My own attitude, not minding when you said I will show you how to brush the teeth. The negative feeling disappeared there and then and I don't know why, but it should be your personal treatment. It can't be anything else. I don't remember exactly what you said or did, but I remember—Strange, me not minding, that I never turned angry. (#1)

Being on the same level when talking.—You can do this in a better way, and you're shown how to do it and there's never a suggestion that you're bad because you can't take better care of your teeth. (#2)

The participants expressed that they appreciated the attention from the dental team. The personal treatment had felt calm and friendly. According to the participants, the dental team showed interest, understanding and caring.

Friendly, interested, yes—simply attention. I think you showed interest and gave me good care. (#8)

#### 3.3.2 A Positive Treatment Result Requires Time

The participants expressed that the time spent, including all parts of the treatment, was somewhat long. They felt it was tiresome to have one's mouth open for such a long time, although they had been told in advance.

You became a little bit tired at the end, but yes, it is fully understandable. Pretty long time, more than an hour. (#9)

The participants mentioned that it was important that the treatment was carefully performed and if the time spent was too short the treatment result would be compromised. It was important to achieve a good treatment result. All participants expressed an understanding for the time it took and said this increased the chances for a positive treatment result. The pauses in between the different parts of the treatment elements were appropriate because they gave them time to recover, relax and ask questions.

Most important is to achieve a good treatment result and then it is necessary to open your mouth for an hour. But there are some pauses in between. (#4)

Pauses were automatically coming in and you could talk about … Then you have the time to relax. (#6)

### 3.4 Comprehensive Care as an Important Factor for a Good and Safe Treatment Experience

This theme was formed when analysing the latent content of the text. Treatment for peri‐implant mucositis was not just the active treatment procedure (in this case, it was performed with a laser or an ultrasonic scaler) but included many more parts. Receiving information about the treatment process before and during the treatment gave a feeling of safe care. The biggest benefit for the participants was being instructed and shown how to perform oral hygiene; repeated feedback increased their motivation and changed oral hygiene behaviours. Moreover, there was an understanding that quality treatment takes time. A personally conducted treatment and being treated with attention and respect were appreciated.

## 4 Discussion

The theme in this study ‘Comprehensive care as an important factor for a good and safe treatment’ indicated that it seemed important to the participants to be treated with a personal approach. This included to being respectfully treated, and being provided with information about the treatment process, open communication and thorough oral hygiene instructions. It also included that their physical comfort was ensured, and their fear and anxiety were relieved during the treatment.

When sitting in the dental chair and being unable to influence what happens the patient can easily feel ‘left out’ and impersonally treated. A personal relationship with the mouth was highlighted, which is not surprising as the mouth is a gateway to the body. For a safe experience of the treatment, it is needed to meet a calm, friendly and informative therapist. These components, that the participants in this study experienced as valuable, are included in person‐centred care, which has been suggested by McCormack et al. [20] as an approach to medical practice, underpinned by values of respect for persons and an individual's right to self‐determination, mutual respect and understanding. Another definition for person‐centred care has been proposed by the International Institute of Medicine (IOM), and includes being respectful of patients' values, preferences and expressed needs; being coordinated and integrated; providing information, communication and education; ensuring physical comfort; providing emotional support and relieving fear and anxiety [21]. The participants' experiences can be interpreted as meaning that person‐centred care was important to them.

In the present study, the participants gained knowledge about the importance of how to brush the teeth so as not to lose their implant. They felt that they had learnt something new, and the greatest benefit of the treatment appears to have been the information and instructions on oral hygiene. The review of oral hygiene was thorough, and the repeated feedback increased the participants' motivation and changed their oral hygiene habits. This result is in concordance with a recent qualitative study, which reported that visual evidence of fewer bacterial deposits on the teeth and less bleeding from the gums encouraged continued behavioural change efforts [22]. However, in a qualitative study by Liss et al. [23] concerning periodontitis, the interviewed dental hygienists commonly did not focus on infection control, although they knew about its importance. Own beliefs regarding the patients' willingness and ability to cooperate, rather than the patients' own preferences, commonly guided the therapy. The dental hygienists wanted to avoid discussions about costs and therefore, as they thought, deleterious effects on their professional relationship with the patient. Similar reasons for avoiding smoking cessation education in patients with periodontitis were shown in a study by Andersson, Westergren and Johannsen [24] Barriers to providing extended information and education in periodontal therapy have been stated to be time and money.

A successful outcome of peri‐implant mucositis therapy includes infection control through patients' oral hygiene measures [25], and therefore, it is necessary that the patient acquires knowledge on how to perform thorough oral hygiene, even if this takes time and costs money. Remarkably, in the present study, some participants said that they had never previously received education on how to perform oral hygiene. As they began to fully understand the importance of oral hygiene previous barriers to performing self‐hygiene were removed. Repeated feedback is vital for entrenching a behaviour and increasing motivation and was highly appreciated by the participants. In the study of adolescents by Dimenäs et al. [22], reminders and support to keep up oral hygiene routines over time were perceived as necessary. Taking the time needed for treatment was not considered a problem by the participants in the present study. Rather, the participants saw it as necessary as good‐quality treatments take time. However, one limitation of our study is that the participants did not pay for the treatment because they participated in a RCT study, which may have had a positive impact on their perception of time.

In our study, most participants expressed no discomfort, regardless of the technique used. A few participants felt discomfort during and a few days after treatment and some reported that the discomfort increased with each treatment session. As far as we know, no other study exists on discomfort in the treatment of peri‐implant mucositis. In a qualitative interview study by Malmqvist et al. [17], it seemed that participants who had been laser‐treated for peri‐implantitis experienced discomfort on the treatment day and up to 2 days after treatment. In the same study, the discomfort was, however, reported to be milder than expected, and the worst part was the local anaesthesia. A limitation in our study may be that the time between treatment for peri‐implant mucositis and the interviews varied from half a month to 6 months, which may have had an impact on the result. The closer the interview was to the treatment, the clearer the experience should have been. For those participants where time had elapsed, elements of the experience may have been lost.

This qualitative study can be considered an important complement to the previous RCT study. The experiences of the treatment were taken into consideration in the design of the interview guide by a focus group interview and not on what our research group thought. This must be considered a strength and should increase the confirmability, since the questions were member‐checked.

A strategic selection of participants for the focus group ensured that the participants were able to express themselves. Of course, there might be a risk that some participants may have difficulties in expressing themselves in groups. However, in the present study, all participants interacted with each other and contributed with their experiences, they reflected on them easily and had a good ability to express themselves. In the interview process, the participants were given enough time to discuss and reflect and hence formed a deeper understanding of the content. This communication enables the expression of experiences, thoughts and ideas to adapt to a person's emotional and information needs [26]. There may, however, be a risk that a strategic selection of participants will favour the ones with the most positive experiences.

Individual interviews were conducted with nine participants, which may seem too few. However, a couple of more interviews were conducted when saturation had arisen, suggesting that no new information would have been added if additional participants had been included.

The first author (V.W.B.) had been clinically treating the participants and a dentist–patient relation between the researcher and the participants was already established before the interviews. This can be seen both as a strength and as a weakness. The researcher having a preunderstanding of the disease itself and its treatment constitutes an advantage in the interview process because of knowledge and experiences. Also, the participants may already feel safe and comfortable and, therefore, be less reluctant to express their thoughts and opinions. The established relation can, however, also pose a risk, shifting focus from what the participants express to the disease itself. In some cases, the participants may have felt uncomfortable and in a position of dependence, especially if they had negative experiences of the treatment.

Both the manifest and the latent content were analysed using content analysis [20]. To strengthen the credibility and dependability of the interpretations, all authors, each with a different preunderstanding, collaborated in the content analysis, that is, using investigator triangulation. In this way, a critical approach and high quality of the interpretations of the interview data were ensured [27]. In addition, the collaboration was interdisciplinary because the authors possess different competencies regarding profession (within and outside dentistry). In this way, the data were analysed from both an inside and an outside perspective that might increase the transferability of the results to similar groups in the treatment of peri‐implant mucositis.

Person centredness is becoming more common in health care policies even though the evidence shows that it is hard to achieve in practice [20]. In Sweden, the national guidelines still do not specifically handle person centredness [28]. Further, person‐centred guidelines are also missing in the European Federation of Periodontology clinical practice guideline on prevention and treatment of peri‐implant diseases [25]. This study emphasises, through the latent findings, the importance of a person‐centred approach. It shows that a dialogue is essential in order to take the person's entire life situation and needs into consideration compared to patient‐centred approach that largely focuses on the disease and functionality [29]. The voice of a person is fundamental for understanding what a good treatment experience is in peri‐implant mucositis. Information and education may take some time but improve motivation and oral hygiene habits. In the treatment of peri‐implant mucositis, comprehensive care appeared to be important for a good and safe patient experience.

## 5 Conclusion

The present study highlights factors of importance in the treatment of peri‐implant mucositis with laser and ultrasonic scaler. A person‐centred approach with respect and attention is important for a good and safe experience and may be important factors in future treatments.

## 6 Clinical Relevance

### 6.1 Scientific Rationale for the Study

There is a lack of studies on the experience of treatment for peri‐implant mucositis.

### 6.2 Principal Findings

Personal treatment provided with respect and attention improves the experience of a good and safe treatment in the treatment of peri‐implant mucositis. Education and hands‐on instructions in oral hygiene improve motivation and change oral habits. Most participants experienced no discomfort in the treatment. In addition, the participants expressed an understanding that quality treatment needs proper time.

### 6.3 Practical Implications

For a good treatment experience in peri‐implant mucositis, it appears that the clinician should strive for a person‐centred approach. Education and repeated feedback on self‐performed oral hygiene may take time but can increase motivation and change habits.

## Author Contributions

Viveca Wallin Bengtsson contributed to the basic conception, design, data acquisition through interviews, data analysis and data interpretation, and drafted and critically revised the manuscript. Pia Andersson contributed to the basic conception, design, data analysis and data interpretation, and drafted and critically revised the manuscript. Kirsti Skovdahl contributed to the basic conception, design, data analysis and data interpretation, and drafted and critically revised the manuscript. All authors have given their final approval and agree to be accountable for all aspects of the work.

## Conflicts of Interest

The authors declare no conflicts of interest.

## Supporting information


Data S1:

Data S2:

## Data Availability

The data that support the findings of this study are available from the corresponding author upon reasonable request.

## References

[1] D. Buser , L. Sennerby , and H. De Bruyn , “Modern Implant Dentistry Based on Osseointegration: 50 Years of Progress, Current Trends and Open Questions,” Periodontology 2000 73 (2017): 7–21, 10.1111/prd.12185. 28000280

[2] S. Meyer , C. Giannopoulou , D. Courvoisier , M. Schimmel , F. Müller , and A. Mombelli , “Experimental Mucositis and Experimental Gingivitis in Persons Aged 70 or Over. Clinical and Biological Responses,” Clinical Oral Implants Research 28, no. 8 (2017): 1005–1012, 10.1111/clr.12912. 27333829 PMC5599942

[3] N. U. Zitzmann , T. Berglundh , C. P. Marinello , and J. Lindhe , “Experimental Peri‐Implant Mucositis in Man,” Journal of Clinical Periodontology 28, no. 6 (2001): 517–523, 10.1034/j.1600-051x.2001.028006517.x. 11350518

[4] R. Pontoriero , M. P. Tonelli , G. Carnevale , A. Mombelli , S. R. Nyman , and N. P. Lang , “Experimentally Induced Peri‐Implant Mucositis. A Clinical Study in Humans,” Clinical Oral Implants Research 5, no. 4 (1994): 254–259, 10.1034/j.1600-0501.1994.050409.x. 7640340

[5] A. M. Roos‐Jansåker , C. Lindahl , H. Renvert , and S. Renvert , “Nine‐ to Fourteen‐Year Follow‐Up of Implant Treatment. Part II: Presence of Peri‐Implant Lesions,” Journal of Clinical Periodontology 33, no. 4 (2006): 290–295, 10.1111/j.1600-051X.2006.00906.x. 16553638

[6] T. Berglundh , G. Armitage , M. G. Araujo , et al., “Peri‐Implant Diseases and Conditions: Consensus Report of Workgroup 4 of the 2017 World Workshop on the Classification of Periodontal and Peri‐Implant Diseases and Conditions,” Journal of Clinical Periodontology 45, no. Suppl 20 (2018): 286–291, 10.1111/jcpe.12957. 29926491

[7] S. Renvert and I. Polyzois , “Treatment of Pathologic Peri‐Implant Pockets,” Periodontology 2000 76, no. 1 (2018): 180–190, 10.1111/prd.12149. 29239086

[8] R. Porras , G. B. Anderson , R. Caffesse , S. Narendran , and P. M. Trejo , “Clinical Response to 2 Different Therapeutic Regimens to Treat Peri‐Implant Mucositis,” Journal of Periodontology 73, no. 10 (2002): 1118–1125, 10.1902/jop.2002.73.10.1118. 12416768

[9] L. J. A. Heitz‐Mayfield and G. E. Salvi , “Peri‐Implant Mucositis,” Journal of Clinical Periodontology 45, no. Suppl. 20 (2018): 237–245, 10.1111/jcpe.12953. 29926488

[10] L. J. A. Heitz‐Mayfield , G. E. Salvi , D. Botticelli , et al., “Anti‐Infective Treatment of Peri‐Implant Mucositis: A Randomised Controlled Clinical Trial,” Clinical Oral Implants Research 22, no. 3 (2011): 237–241, 10.1111/j.1600-0501.2010.02078.x. 21251076

[11] M. Thöne‐Mühling , K. Swierkot , C. Nonnemacher , R. Mutters , L. Flores‐de‐Jacoby , and R. Mengel , “Comparison of Two Full‐Mouth Approaches in the Treatment of Peri‐Implant Mucositis: A Pilot Study,” Clinical Oral Implants Research 21, no. 5 (2010): 504–512, 10.1111/j.1600-0501.2009.01861.x. 20128831

[12] R. Renvert , A. M. Roos‐Jansåker , and N. Claffey , “Non‐Surgical Treatment of Peri‐Implant Mucositis and Peri‐Implantitis: A Literature Review,” Journal of Clinical Periodontology 35, no. 8 Suppl (2008): 305–315, 10.1111/j.1600-051X.2008.01276.x. 18724858

[13] A. Aoki , K. M. Sasaki , H. Watanabe , and I. Ishikawa , “Lasers in Nonsurgical Periodontal Therapy,” Periodontology 2000 36 (2004): 59–97, 10.1111/j.1600-0757.2004.03679.x. 15330944

[14] A. Insua , A. M. H. L. Wang , and M. Inglehart , “Patient‐Centered Perspectives and Understanding of Peri‐Implantitis,” Journal of Periodontology 88, no. 11 (2017): 1153–1162, 10.1902/jop.2017.160796. 28548884

[15] J. Derks , Y. Ichioka , C. Dionigi , et al., “Prevention and Management of Peri‐ Implant Mucositis and Peri‐Implantitis: A Systematic Review of Outcome Measures Used in Clinical Studies in the Last 10 Years,” Clinical Periodontology 50, no. Suppl 25 (2023): 55–66, 10.1111/jcpe.13608. 35246865

[16] K. H. Abrahamsson , J. L. Wennström , T. Berglundh , and I. Abrahamsson , “Altered Expectations on Dental Implant Therapy; Views of Patients Referred for Treatment of Peri‐Implantitis,” Clinical Oral Implants Research 28, no. 4 (2017): 437–442, 10.1111/clr.12817. 26918305

[17] S. Malmqvist , J. Erdenborg , G. Johannsen , and A. Johannsen , “Patient's Experiences of Dental Implants, Peri‐Implantitis and Its Treatment—A Qualitative Interview Study,” International Journal of Dental Hygiene 22 (2024): 530–539, 10.1111/idh.12683. 37093891

[18] R. A. Kreuger and M. A. Casey , Focus Groups. A Practical Guide for Applied Research, 5th ed. (Thousand Oakes, CA: Sage Publications, 2014).

[19] U. H. Graneheim and B. Lundman , “Qualitative Content Analysis in Nursing Research: Concepts, Procedures and Measures to Achieve Trustworthiness,” Nurse Education Today 24, no. 2 (2004): 105–112, 10.1016/j.nedt.2003.10.001. 14769454

[20] B. McCormack , S. Dulmen , H. Eide , K. Skovdahl , and T. Eide , Person‐Centred Healthcare Research (Hoboken, NJ: John Wiley & Sons Ltd., 2017).

[21] Institute of Medicine (IOM) , Crossing the Quality Chasm: A New Health System for the 21st Century (Washington: National Academy Press, 2001). 25057539

[22] S. L. Dimenäs , A. L. Östberg , M. Lundin , J. Lundgren , and K. H. Abrahamsson , “Adolescents' Experiences of a Theory‐Based Behavioural Intervention for Improved Oral Hygiene: A Qualitative Interview Study,” International Journal of Dental Hygiene 20, no. 4 (2022): 609–619, 10.1111/idh.12606. 35925040 PMC9804348

[23] A. Liss , K. Nilsson , J. L. Wennström , and K. H. Abrahamsson , “Complexity of Best‐Evidenced Practice in Periodontal Therapy: Views of Swedish Dental Hygienists,” International Journal of Dental Hygiene 18, no. 3 (2020): 220–227, 10.1111/idh.12431. 32069383

[24] P. Andersson , A. Westergren , and A. Johannsen , “The Invisible Work With Tobacco Cessation—Strategies Among Dental Hygienists,” International Journal of Dental Hygiene 10, no. 1 (2012): 54–60, 10.1111/j.1601-5037.2011.00530.x. 21974714

[25] D. Herrera , T. Berglundh , F. Schwarz , et al., “EFP Workshop Participants and Methodological Consultant. Prevention and Treatment of Peri‐Implant Diseases—The EFP S3 Level Clinical Practice Guideline,” Journal of Clinical Periodontology 50, no. Suppl 26 (2023): 4–76, 10.1111/jcpe.13823. 37271498

[26] H. Eide and T. Eide , Kommunikasjon i Relasjoner: Samhandling, Konfliktlösning, Etikk, [in Norweigan], 2nd. Rev. Og utv. Utg. ed. (Oslo, Norway: Gyldendal Akademisk, 2017).

[27] H. Noble and H. Roberta , “Triangulation in Research, With Examples,” Evidence‐Based Nursing 22, no. 3 (2019): 67–68, 10.1136/ebnurs-2019-103145. 31201209

[28] National Board of Health and Welfare , Nationella Riktlinjer för Tandvård. Stöd för Styrning Och Ledning 2022 [in Swedish] (Stockholm, Sweden: Socialstyrelsen, 2022), https://www.socialstyrelsen.se/globalassets/sharepoint‐dokument/artikelkatalog/nationella‐riktlinjer/2022‐9‐8114.pdf.

[29] J. Håkansson Eklund , I. K. Holmström , T. Kumlin , et al., “‘Same Same or Different?’ A Review of Reviews of Person‐Centered and Patient‐Centered Care,” Patient Education and Counseling 102, no. 1 (2019): 3–11, 10.1016/j.pec.2018.08.029. 30201221

